# SOCIALLY WITHDRAWN OR SOCIALLY ENGAGED? THE IMPACT OF GRANDPARENTING ON SOCIAL PARTICIPATION AMONG OLDER ADULTS

**DOI:** 10.1093/geroni/igae098.1485

**Published:** 2024-12-31

**Authors:** Jason Wong, Mengke Zhao, Yuying Tong, Feinian Chen

**Affiliations:** Yale University, New Haven, Connecticut, United States; The Chinese University of Hong Kong, Hong Kong, China (People’s Republic); The Chinese University of Hong Kong, Hong Kong, China (People’s Republic); Johns Hopkins University, Baltimore, Maryland, United States

## Abstract

**Objectives:**

Grandparents caring for grandchildren (i.e., grandparenting) is an important aspect of intergenerational family dynamics in the 21st Century. Despite extensive literature documenting its impact on grandparents’ health, the mechanisms remain insufficiently addressed, particularly those related to social support and engagement. Grandparenting may promote role enhancement and create opportunities for social participation, but it may also result in role strain and reduce time availability for social activities, depending on caregiving intensity and living arrangements. This study addresses this gap by examining the effect of grandparenting on grandparents’ social participation and its gendered implications.

**Methods:**

Data are from 2011, 2013, and 2018 waves of the China Health and Retirement Longitudinal Study (11,547 older adults aged 50-84). Individual-level fixed-effects linear probability models are employed. Grandparenting is assessed through caregiving intensity and living arrangements. We measure both formal social participation (FSP, e.g., club activities or volunteering) and informal social participation (ISP, e.g., meeting or helping friends).

**Results:**

All types of grandparenting, except those under the three-generation living arrangement, increase grandparents’ ISP. Gender differences are observed. Compared to no grandparenting, providing noncoresident nonintensive care and skipped-generation care increases ISP only for grandfathers, while providing noncoresident intensive care and three-generation care increases ISP only for grandmothers. We find no association between any type of grandparenting and FSP.

**Discussion:**

Overall, grandparenting facilitates rather than deters social connectedness for grandparents. This study underscores the importance of intergenerational linked lives and family networks in maintaining social connectedness among grandparents. The gendered division of grandparenting responsibilities is also discussed.

